# Recurrent retroperitoneal liposarcoma: A case report and literature review

**DOI:** 10.1002/ccr3.4717

**Published:** 2021-08-30

**Authors:** Francesk Mulita, Georgios‐Ioannis Verras, Elias Liolis, Levan Tchabashvili, Dimitrios Kehagias, Charalampos Kaplanis, Ioannis Perdikaris, Ioannis Kehagias

**Affiliations:** ^1^ Department of General Surgery General University Hospital of Patras Patras Greece; ^2^ Department of Internal Medicine General University Hospital of Patras Patras Greece

**Keywords:** liposarcoma, recurrence, retroperitoneal

## Abstract

Retroperitoneal liposarcoma frequently recurs within 2 years of the initial surgical resection. For the early detection of recurrent retroperitoneal liposarcomas, a shorter follow‐up interval with CT or MRI would be helpful.

## Graphical abstract

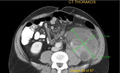

## 1 INTRODUCTION

This report describes the case of a patient diagnosed with a local recurrent retroperitoneal liposarcoma after complete tumor resection 30 months ago. Magnetic resonance imaging of the abdomen was used to evaluate the tumor. The recurrent mass was about 19 cm and the patient underwent reoperation.

Soft‐tissue sarcomas (STS) are less than 1% of all malignant tumors in adults. Liposarcoma is the most common variant and accounts for approximately 15% of adult soft‐tissue tumors.^1^ The annual incidence of soft‐tissue sarcomas is approximately 2–5 per 100,000 per year.^2^ Retroperitoneal liposarcoma is the most common case that represents 40% of all soft‐tissue sarcomas that occur in the retroperitoneum.^3^, ^4^ Due to the large retroperitoneal space, patients with retroperitoneal liposarcoma have obvious symptoms at a very late stage, when the mass develops enough to press or invade the neighboring organs. Because of the late diagnosis of this tumor, there is a low rate of complete resection of this malignancy. In addition, there is a higher rate of recurrence compared with liposarcomas in other parts of the human.^1^, ^5^

## 2 CASE REPORT

**FIGURE 1:**
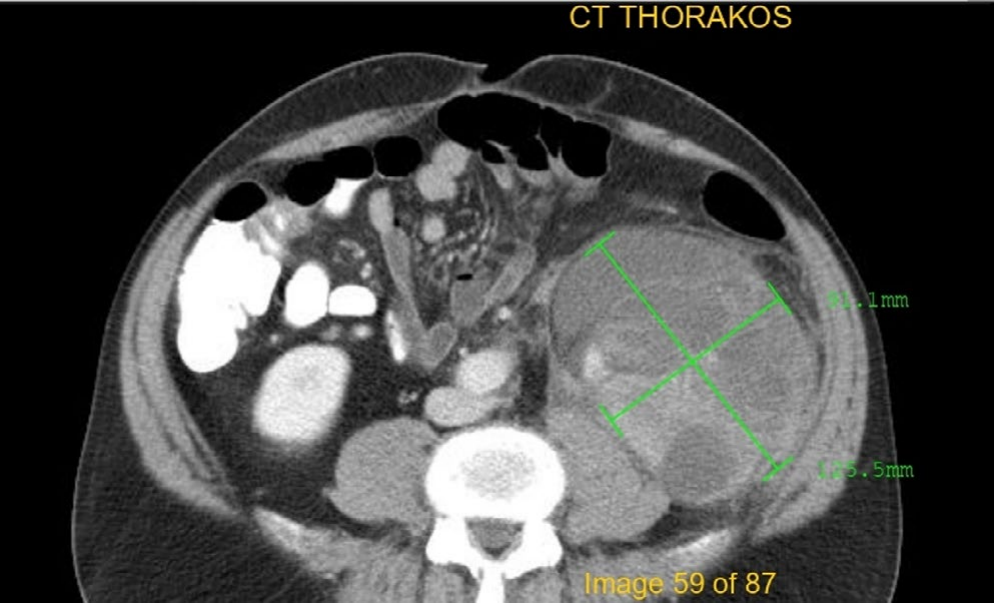
Sixty‐two‐year‐old man with recurrent retroperitoneal liposarcoma; CT scan shows a soft‐tissue mass in retroperitoneum. The longest diameter is 12.5 cm

**FIGURE 2:**
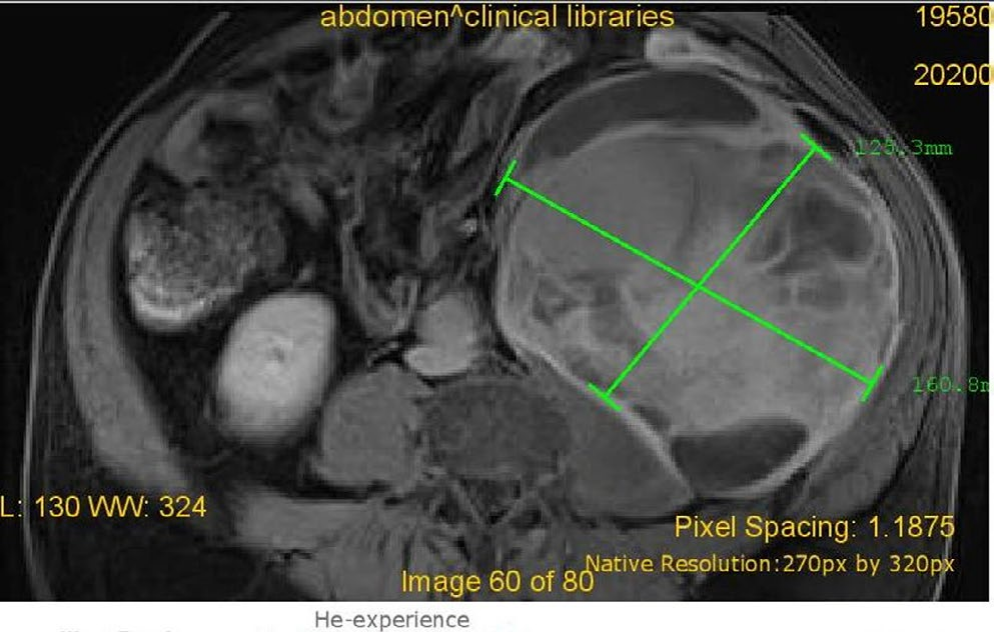
Sixty‐two‐year‐old man with recurrent retroperitoneal liposarcoma; MRI 2 months after the CT scan. The mass has increased in size about 4 cm

**FIGURE 3:**
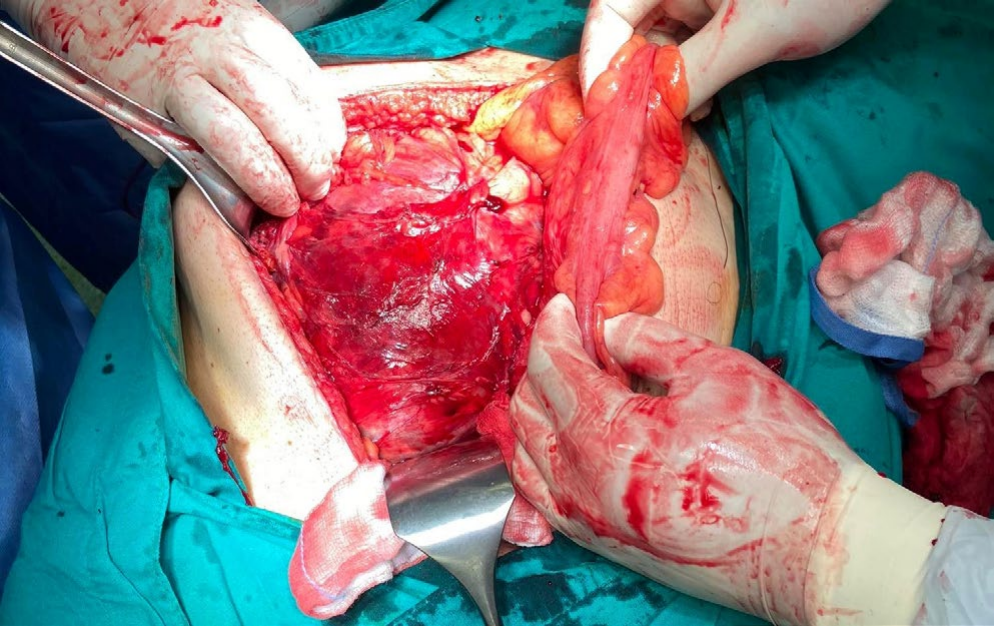
Sixty‐two‐year‐old man with recurrent retroperitoneal liposarcoma; Laparotomy with midline incision was approached. The resection of the retroperitoneal mass was 19 × 17 × 16 cm

A 62‐year‐old man was admitted to our department in June 2017 with abdominal pain and urinary difficulties. A retroperitoneal mass measuring 22 × 15 × 12 cm was observed in an abdominal computed tomography (CT). However, the tumor has not spread to the lymph nodes or other parts of the body. The patient underwent an excision of the tumor. The histopathological examination presented a well‐differentiated (grade I) liposarcoma that weighed 1172 g and measured 25 × 18 × 13 cm (stage IIIB according to American Joint Committee on Cancer TNM system). Because of microscopic positive margins, the patient received adjuvant chemotherapy (doxorubicin, ifosfamide) for eight cycles and complete remission was demonstrated by an abdominal CT follow‐up after the 8th cycle. The patient's clinical follow‐up by oncology group and imaging scans showed no evidence of recurrence 12 months after the 8th cycle of chemotherapy. After treatment, routine follow‐up continued on regular basis with physical examination, abdominal ultrasound (US) every 3 months, and CT every 12 months. In October 2019, the patient had the abdominal US, but no mass was detected. In December 2019, the 62‐year‐old man got readmitted because of a complaint of pain in the back of the left hip. The pain was moderate in severity. On examination, it was also revealed mild back pain as well as a palpable abdominal mass. Bowel sounds were audible, and rectal examination was normal. His vital signs were unremarkable. His routine blood tests including hemogram, C‐reactive protein level, liver, and renal function test, serum amylase, and lipase were normal. Chest and abdominal radiography showed no abnormalities. He was submitted to a CT (Figure 1) showing a retroperitoneal mass, measuring 12.5 × 10.1 × 9.2 cm. In February 2020, the patient had magnetic resonance imaging (MRI; Figure 2) in which the mass was increased 4 cm in size. PET‐CT was also performed and showed no other abnormality. In March of the same year, he underwent a new excision of the tumor (Figure 3) and the histopathological examination presented a low‐grade liposarcoma. The patient was discharged and referred to the oncology department for further management.

## 3 DISCUSSION

Liposarcoma (LPS) is the most common histological subtype of soft‐tissue sarcomas (STS), a family of mesenchymal‐derived malignancies that measures over 50 different histological subtypes.^6^ They account for just over 20% of the STS encountered and are further classified into four principal subtypes: well‐differentiated (WD), dedifferentiated (DD), myxoid (MD), and pleomorphic (PL) liposarcomas.^7^ LPS can be found throughout the body, with the most common localizations, being in the extremities, head and neck region, truncal wall, retroperitoneum, and mesentery.^8^ WD and DD liposarcomas account for the majority of the cases found in literature and comprise about 50% of the total liposarcoma incurrences.^7^ In fact, as their name implies, WD liposarcomas seem to be the precursor of DD LPS, since their histological appearance is very similar, except for DD LPS, having characteristics of higher‐grade tumors, more dense cellular distribution, and more intense staining, for target carcinogenesis‐related molecules, such as MDM2, CDK4, YEATS4, and CPM, all presumed to play a central role in the loss of differentiation, and progression from WD to DD liposarcomas.^7^, ^9^, ^10^ The presence of distinct areas of non‐adipogenic sarcomatous tissue is the histopathological characteristic of DD LPS.^7^, ^9^, ^11^ Both WD and DD seem to have poor responses to either chemotherapy or radiotherapy, in contrast with the myxoid and pleomorphic subtypes, that are deemed as good responders.^12^, ^13^, ^14^ Among them, pleomorphic LPS is the most aggressive subtype, consisting mainly of lipoblasts of high‐grade morphology, without resembling any known cell lineage, like the other subtypes.^7^

Treatment of primary LPS, according to the latest guidelines, must always involve a multidisciplinary team of practitioners, and all the patients should be referred to physicians with experience in STS management, if possible. According to the NICE recommendations for the treatment of sarcoma patients, centralizing the treatment of such patients was deemed so necessary that led to the formation of 15 specialized centers for the management of sarcoma.^15^, ^16^ The teams' composition should include radiologists, surgeons, and medical and clinical oncologists, with consultations from plastic, vascular, chest, and neurological surgeons, depending on the tumor's site (e.g., sarcomas infiltrating major nerve structures, or surface liposarcomas requiring extensive reconstruction).^6^, ^15^ In most patients, staging of the suspected LPS requires preoperative ultrasound assessment (depending on accessibility), MRI imaging, and core‐needle biopsy retrieval.^9^, ^15^ Surgical resection of the malignancy remains the treatment of choice for liposarcomas, according to most major international societies' guidelines.^6^, ^15^, ^16^, ^17^ Resection with clinically negative margins (R0) is the approach of choice, if feasible. According to the latest ESMO classification of surgical margins in oncological surgery, R0 resection is typically achieved with wide (the surgical plane in healthy tissue and same compartment as the tumor) or radical (removal of affected compartments) excisions of liposarcomas.^17^ While there is no current official consensus, a macroscopically healthy margin of 1 cm around the primary tumor is considered an adequate, R0 excision. After the tumor's resection, local radiotherapy usually follows for grade 2/3, >5 cm, and deep lesions.^6^, ^17^ Adjuvant radiation treatment administration is highly effective in local recurrence prevention, preservation of functionality as well as has similar overall survival when compared to radical excision.^18^ Radiotherapy may be remitted in select patients with superficial and small (<5 cm) tumors that underwent adequate R0 excision.^6^, ^17^ Some panels have concluded that preoperative instead of postoperative radiation seems to have comparable results, while using a smaller radiation field, with the proposed benefit of pseudocapsule fibrosis and shrinkage around the tumor, presumably preventing intraoperative seeding.^6^, ^19^, ^20^ Planned R1 excision is also an acceptable option when wide margin resection is not feasible; it is, however, followed by radiation treatment, except for stage IA patients, where close monitoring is also an option.^6^ Re‐excision, after confirmation of unplanned positive margins, is the preferred approach for patients with an adequate functional status that are good surgical candidates, with minimal predicted effect on morbidity. Compartectomy, especially in limb liposarcomas, is a procedure that frequently supplements an initial R2 excision, with good survival outcomes, provided that a reconstruction specialist is part of the multidisciplinary team that manages the patient.^12^, ^16^ One point of conflict, in the management of localized, resectable sarcomas, is the presence of metastatic lymph node disease. While total resection remains an option, some advocate the enrollment of such patients in preoperative chemotherapy protocols, or more often, administrating postoperative chemoradiation therapy, lacking, however, formal consensus on the matter.^19^ Use of neoadjuvant treatment, either chemotherapy or radiotherapy, is somewhat controversial, for liposarcoma, and is currently indicated for borderline unresectable tumors depending on the reported chemosensitivity of each subtype, with the goal of downstaging the tumor to resectable status.^6^, ^17^, ^19^ Preoperative radiotherapy is more commonly used for downstaging purposes, and neoadjuvant chemotherapy is mostly reserved for stage III/IV, high‐grade disease, or large tumors.^6^, ^15^, ^16^ MD and round cell liposarcomas are prime candidates for chemotherapy induction, PL liposarcomas have been characterized as moderately sensitive, and DD and WD are on the chemoresistant end of the spectrum.^6^, ^15^, ^16^, ^19^ Regarding advanced, or unresectable disease, the most widely accepted approach is that of initiation of doxorubicin.^19^ Palbociclib has been recommended for unresectable retroperitoneal, WD liposarcomas.^6^ Unresectable retroperitoneal or intra‐abdominal disease follows the same recommendations, except for palliative surgery being performed far more often, due to disease complications.^6^, ^9^, ^16^, ^19^, ^21^

Recurrence in liposarcoma patients is frequent. Approximately 50% of patients with grade II/III liposarcomas will develop either local disease recurrence or distant metastatic disease, with a median survival of 12 months.^22^ In total, up to 24% of the patients with liposarcoma will have recurrent disease, no matter the grade or subtype, and 70% of the patients with a retroperitoneal liposarcoma will die from recurrence‐related adverse effects.^8^, ^9^ Risk assessment of patients with primary liposarcoma, concerning future relapses, must include tumor grade, tumor size, and histological subtype.^13^, ^14^, ^23^ Complete tumor resection, preferably on a single R0 excision, is the primary prognostic factor, affecting disease recurrence.^6^, ^23^, ^24^, ^25^ Administration of postoperative chemoradiation was also shown to have favorable effects on local and distant recurrence, as well as recurrence‐free survival in patients with extremity sarcomas.^26^ A 2018 meta‐analysis on liposarcoma recurrence identified histological subtype as a major prognostic factor for both local recurrences, and distant metastases, with DD and PL liposarcomas, having the worst recurrence percentages.^13^ Male gender and age at the time of diagnosis were also found to negatively correlate to overall survival, and recurrence‐free survival.^13^ A retrospective study on recurrent retroperitoneal liposarcoma recently showed that between all subtypes, DD liposarcoma had the earliest mean recurrence time, measuring at 0.9 years from first diagnosis.^9^ Incomplete resection, total unresectability of the tumor, and high‐grade tumors are identified as the major risk factors for recurrence, concerning retroperitoneal sarcomas.^27^ A 2009 study of 105 patients also showed that once local recurrence has occurred, the growth rate of the tumor is a major survival prognostic factor, with growth rates of above 0.9 cm/month being an ominous sign, despite appropriate surgical management.^28^ Late local recurrence is also a reality in patients diagnosed with liposarcoma since studies have shown that up to 14% of the patients treated for sarcoma of any histology, with tumor size >10 cm, had recurrent disease after 5 years, with diagnoses of relapse being made up to 10 years postoperatively.^24^ To detect local or distant relapses early, close monitoring of patients is needed, especially utilizing MRI and CT chest scans, since lung metastasis is the most common distant relapse site. ESMO currently suggests follow‐up every 3–4 months for the first 2–3 years, then twice the fifth year, and annually after that, for patients with high/intermediate grade disease.^19^

For the treatment of liposarcoma recurrence, the primary contributing factor is the type of recurrency: local relapse of the disease, or distant metastatic lesions. Whenever a patient presents with local recurrent liposarcoma, the attending team of physicians must first thoroughly evaluate for the presence of concurrent distant metastasis, usually utilizing PET‐CT scans, or MRI, before further deciding upon treatment.^6^, ^8^, ^15^, ^29^ Local recurrence alone is recommended to be managed surgically, with repeat lesion surgery, performing R0 excision if the patient's overall status permits such operations.^6^, ^17^, ^21^ In recurrent liposarcomas of the trunk and extremities, such operations often result in amputating states, and the possibility, impact, and rehabilitation from those approaches must be discussed with the patient.^6^ Some authors also claim that patients with significant comorbidities, presenting with local relapse alone, of WD liposarcomas, can be managed with close monitoring of the disease, rather than surgery.^30^ Irradiation after re‐excision is recommended as the standard of care for patients naïve to radiation therapy, with the additional option of preoperative instead of postoperative radiation, still in play.^6^ Re‐irradiation of the affected site, in patients having received radiotherapy for primary tumors, does not seem to have a clear consensus, is decided on a patient by patient basis, and most often given in the form of brachytherapy.^6^, ^19^, ^31^ In the event of regional nodal metastases found to accompany the regional recurrence, options include metastasectomy with regional lymphatic excision (where possible), followed by chemotherapy, localized brachytherapy irradiation protocols, and isolated limb perfusion techniques followed by surgery.^17^, ^26^, ^31^ The discovery of disseminated metastases, away from the primary tumor site, is most often treated with the administration of chemotherapy.^17^ Commonly used regimens include doxorubicin, epirubicin, ifosfamide, liposomal doxorubicin, and, most recently approved, the CDK 4 and 6 kinase inhibitor palbociclib.^6^, ^27^, ^32^ The same principles of primary tumor treatment apply to retroperitoneal liposarcoma recurrence, dividing the disease once again, in resectable and unresectable. Limited data support that patients suitable for recurrent retroperitoneal liposarcoma excision should first undergo induction radiotherapy if it was not part of their primary tumor treatment. Patients desensitized to radiation, from previous treatment, should receive neoadjuvant chemotherapy, based on the regimens mentioned above.^6^, ^27^, ^33^ Postoperative patients recovering from recurrent intra‐abdominal liposarcoma surgery are not recommended to undergo chemotherapy if R0 surgical margins are confirmed.^6^, ^31^ In the case of R1 or R2 margins, chemotherapy regimens are superior to either regional irradiation or brachytherapy.^6^ Surgery in unresectable and metastatic disease is the only palliative in nature and is only undertaken in patients with recurring intra‐abdominal liposarcomas that are symptomatic, aiming toward quality‐of‐life improvement.^6^, ^31^

Histological subtypes must once again be incorporated in the decision‐making, about patients with recurrent liposarcoma. Myxoid and clear cell histology have greater metastatic potential than that of the WD or DD and therefore should be monitored in tissues other than the chest region alone, for example, spine, pelvis, and fat pads.^14^, ^25^, ^30^ Insensitivity to either chemotherapy or radiation means that recurrences of those subtypes can be managed with multiple repeat debulking surgeries.^30^ Trabectedin has been used in clinical trials for disease recurrence and has shown promising results in the overall survival of MD liposarcoma patients.^30^ Pleomorphic liposarcoma, on the other hand, is chemo‐sensitive, and therefore, the administration of neoadjuvant chemotherapy, or chemotherapy alone (in unresectable recurrences), is the preferred method of treatment.^30^, ^32^, ^33^

## 4 CONCLUSION

The retroperitoneal liposarcoma is a rare type of malignancy, and surgery is considered the first‐line treatment. The diagnosis of well‐differentiated retroperitoneal liposarcoma and postoperative follow‐up of patients is very difficult. Relapse after surgery is very frequent and shorter follow‐up interval with CT or MRI would be helpful, in order to detect the tumor earlier.

## CONFLICT OF INTEREST

None declared.

## AUTHOR CONTRIBUTION

FM, G‐IV, EL, LT, DK, IP, and CK contributed to the clinical data collection and prepared the case report. FM and IK contributed to the design of the case report presentation and performed the final revision of the manuscript.

## Data Availability

Data available on request from the authors.
